# A quality improvement (QI) project on improving trainee confidence in conducting remote psychiatric consultations at Pennine Care National Health Service (NHS) Foundation Trust in the United Kingdom (UK)

**DOI:** 10.1192/bjo.2021.560

**Published:** 2021-06-18

**Authors:** Rachel Moir, Roshelle Ramkisson, Seri Abraham, Shevonne Matheiken

**Affiliations:** 1 Health Education England North West; 2 Pennine Care NHS Foundation Trust; 3 Northamptonshire Healthcare NHS Foundation Trust

## Abstract

**Aims:**

When the coronavirus disease 2019 pandemic hit the UK, clinicians within Pennine Care NHS Foundation Trust (a five-borough mental health trust) were faced with the challenge of rapidly switching to a novel way of assessing patients remotely.

The idea for a QI project on trainees’ experience with remote consultations was conceived in April 2020. We present our February 2021 results here.

We aimed to improve trainee confidence in conducting remote psychiatric assessments by at least 40%, to ensure effective and safe patient care during their 6 months placement.

**Method:**

Our discovery process included surveying trainees in April 2020 to explore experiences with remote psychiatric consultations, a literature search of current UK guidance and a local audit. The audit reviewed documentation of consent to remote consultations, with reference to standards as per NHS England remote consultation guidance. Key change ideas included publication of an article, ‘Remote consultations – top tips for clinical practitioners’, video-simulated remote consultations and a session on remote consultations in the trainee induction.

In the first ‘plan-do-study-act’ (PDSA) cycle, we presented key findings from the article in a video presentation, which was sent trust-wide. We measured confidence in conducting remote assessments pre- and post-presentation via a feedback survey. Unfortunately, response rates were low and in the second PDSA cycle we targeted a smaller cohort of trainees at the August 2020 induction, although encountered similar difficulties. In the third PDSA cycle, we collected real-time data using an interactive app at the February 2021 trainee induction, and measured pre- and post- confidence following a presentation and a video-simulated remote consultation.

**Result:**

2/34 respondents had accessed previous remote psychiatric consultation training and12/35 had some telepsychiatry experience. Pre-induction trainee confidence results revealed: extremely uncomfortable (16%), not confident (31%), neutral (47%), confident (6%) and very confident (0%) and post-induction confidence was 0%, 22%, 52%, 26% and 0%, respectively.

**Conclusion:**

Our project started during the first peak of the pandemic, which may be a reason for initial limited response rates. Our results suggest that the remote psychiatric consultation trainee induction session has shown some improvement in trainee confidence; the ‘confident’ cohort improved from 6% to 26%.

Our next steps include collecting similar real-time data, mid-rotation and uploading video-simulated remote consultations to the Trust Intranet. We plan to complete the local audit cycle. We also plan to incorporate patient experience (from an ongoing systematic review) to inform a potential triage process post-pandemic, choosing between face-to-face versus remote consultations.

